# *Hirsutella sinensis* Treatment Shows Protective Effects on Renal Injury and Metabolic Modulation in db/db Mice

**DOI:** 10.1155/2019/4732858

**Published:** 2019-04-04

**Authors:** Zhenyao Lu, Sijia Li, Runbin Sun, Xue Jia, Chen Xu, Jiye Aa, Guangji Wang

**Affiliations:** Jiangsu Provincial Key Laboratory of Drug Metabolism and Pharmacokinetics, China Pharmaceutical University, Nanjing 210009, China

## Abstract

*Hirsutella sinensis* (HS) is the anamorph of the traditional Chinese medicine* Cordyceps sinensis*. Although the renal protective effect of HS has been reported, its effect on diabetic nephropathy (DN) remains unclear. In this study, db/db mice were used as the DN model, and the renal protective effect was evaluated after oral administration of HS for 6 and 12 weeks. Plasma, urine, and kidney samples were collected, and biochemical indicator measurements, pathological analysis, and metabolomics studies were performed. Biochemical assays showed that HS reduced the levels of fasting blood glucose (FBG), urinary albumin/creatinine ratio (ACR), and N-acetyl-beta-D-glucosaminidase (NAG) and increased the creatinine clearance (Ccr). HS alleviated glomerular and tubular glycogen accumulation and fibrosis and normalized the disordered ultrastructure of the glomerular filtration barrier. Metabolomics analysis of metabolites in the plasma, urine, and kidney indicated that HS modulated the perturbed glycolipid metabolism and amino acid turnover. HS reduced the elevated levels of metabolites involved in energy metabolism (TCA cycle, glycolysis, and pentose phosphate pathway) and nucleotide metabolism (pyrimidine metabolism and purine metabolism) in the kidneys of db/db mice. These results suggest that HS can protect against renal injury and that its efficacy involved metabolic modulation of the disturbed metabolome in db/db mice.

## 1. Introduction

Diabetic mellitus is an increasing global health challenge and severely affects patients' longevity and quality of life due to hyperglycemia and various complications [1]. It is a chronic metabolic disorder as a result of hereditary and environmental factors [2]. As a common microvascular complication of diabetic mellitus, diabetic nephropathy (DN) is the leading cause of end stage renal disease (ESRD) and is accompanied by high mortality [3]. The pathologic process of DN is very complicated. Briefly, under hyperglycemia and metabolic disturbance, the glomerular capillary and tubular structure and function are altered [4]. For normal glomeruli, a strong glomerular filtration barrier is comprised of the fenestrated endothelial cell layer, basement membrane, and podocytes and is impermeable to macromolecular proteins such as albumin [5]. For healthy kidney tubules, the reabsorption is crucial to recycling energy substrates and water [6, 7]. In diabetes, hyperglycemia causes the excessive generation of advanced glycation end products (AGEs) in renal tissue and then results in the accumulation of extracellular matrix (ECM) and the release of profibrotic cytokines, such as transforming growth factor *β*1 (TGF-*β*1), connective tissue growth factor (CTGF), and the angiogenic growth factor (VEGF), which ultimately leads to renal fibrosis and inflammation [8]. These changes finally lead to glomerular filtration barrier damage and mesangial and tubulointerstitial expansion or hypertrophy [9]. In addition, studies have indicated that metabolic perturbation was involved in the process of DN, such as TCA cycle [10] and purine and pyrimidine metabolism [11]. DN is always accompanied by elevated albuminuria, renal fibrosis, and metabolic disorder.

Unfortunately, clinical treatment of DN remains a challenge due to its complex etiology [12]. Clinically, DN has long been treated with classic hypoglycemic drugs and angiotensin-converting enzyme (ACE) inhibitors/angiotensin II receptor blockers (ARBs) [13]. Undeniably, these drugs can significantly reduce fasting blood glucose and urinary albumin, but the severe renal toxicity and other serious side effects still deserve attention [14, 15]. For example, metformin is the main first-line oral drug of choice in the management of hyperglycemia, but long-term administration of metformin may cause gastrointestinal disturbances and ketoacidosis [16, 17]. Therefore, some new ideas and choices are needed for the treatment of DN. Traditional Chinese Medicines (TCMs) have been widely applied as therapeutic alternatives owing to their good bioactivity and low side effects [18].* Cordyceps sinensis* (CS), commonly called ‘Dong-Chong-Xia-Chao', is an entomopathogenic fungus that has been widely used as a TCM in China for over 300 years [19, 20]. In recent years, many studies have indicated that the kidney protection of CS is mediated by downregulating TGF-*β*1/Smad signaling and weakening renal fibrosis [21, 22]. Due to the increasing demand, the wild resource of CS has been drying up [9]. Thus, development of an alternative that is reliable and economical is necessary.* Hirsutella sinensis* (HS), an anamorph of CS [23], has been artificially cultured on a large scale [24]. The bioactivity of HS is similar to CS because of their analogous composition [25, 26]. HS has been shown to have therapeutic effects on renal function damage by aristolochic acid [27]. However, the renal protection of HS on DN is still unknown.

In this study, db/db mice, a classical type 2 diabetes model [28], were used as the DN model and exposed to HS. Meanwhile, rhein, a therapeutic agent for hyperglycemia in diabetes [29], was chosen as a positive control. Routine biochemical assays and renal pathological changes were evaluated. The metabolites in the plasma, urine, and kidneys of db/db mice were analyzed using a multimetabonomic platform combined with gas chromatography-mass spectrometry (GC-MS) and high-performance liquid chromatography-triple-time-of-flight mass spectrometry (HPLC-QTOF/MS). We aimed to evaluate the protective effects of HS on renal injury and metabolic disorders in db/db mice.

## 2. Materials and Methods

### 2.1. Preparation of* Hirsutella Sinensis* and Rhein Mixed in Normal Chow

The commercial* Hirsutella sinensis* (Lot: 1606127) was provided by Huadong Pharmaceutical Co., Ltd. (Hangzhou, China). The micronized rhein, purified by alkali extraction and acid precipitation as reported [30], was provided by Dr. Zhihong Liu (Nanjing University School of Medicine, Nanjing, China). Preparation of HS and rhein mixed in normal chow was performed by Trophic Animal Feed High-tech Co., Ltd. (Nantong, China). The special chow with 2% HS was composed of 200 grams HS powder in 10 kilograms normal chow (China Experimental Animal Food Standard, GB 14924.2-2001, and GB 14924.3-2001), and the special chow with 0.1% rhein was composed of 10 grams rhein powder in 10 kilograms normal chow. The dosages were determined by the clinical dose.

### 2.2. Animal Experiments

Five-week-old male db/db diabetic mice (n=48) with a C57BL/Ks background and the nondiabetic littermate control db/m mice (n=16) were purchased from Cavens Laboratory (Changzhou, China; Certificate no. SCXK 2016-0010). After one week of acclimatization, db/m mice were randomly divided into two groups (n=8 per group) fed normal chow for 6 weeks and 12 weeks as control group. Db/db mice were randomly divided into six groups (n=8 per group), including two model groups fed normal chow, two HS groups fed 2% HS, and two R groups fed 0.1% rhein (positive control group) for 6 weeks and 12 weeks. Mice were housed in a room at a constant temperature (ca. 22±2°C) and humidity (ca. 60%±2%) with 12 h light/dark cycles. All mice were allowed free access to chow and water except as otherwise noted. The body weight and the ingestion of chow were recorded. Mice were placed individually into metabolic cages for the collection of 24-hour urine before being killed. All urine samples were immediately centrifuged at 4,000 rpm for 10 min to remove precipitation, and the volumes of 24-hour urine were recorded. The mice were fasted overnight, and then, fasting blood glucose (FBG) values were measured with a glucometer (Accu Check Performa, Roche Diagnostic, Nanjing, China) by tail vein. After that, the mice were sacrificed under anesthesia by intraperitoneal injection of avertin (7.2 mg/20 g body weight). Blood samples were collected into the heparinized tubes via the retro-orbital sinus and centrifuged at 8,000 rpm for 10 min to obtain plasma. The plasma and urine were stored at -80°C for further assessment of biochemical parameters and metabonomics. Then, the mice were perfused with normal saline through the heart with a peristaltic pump (Anachem, Luton, UK), and the right kidney was removed with 4% paraformaldehyde solution (Santa Cruz Biotechnology; Santa Cruz, CA) for histopathological analysis or with 2.5% glutaraldehyde solution (Sigma-Aldrich, USA) for ultrastructure analysis. The left kidney was frozen in liquid nitrogen for RT-PCR and metabonomics analyses. The experimental protocols were approved by the Animal Ethics Committee of China Pharmaceutical University (Nanjing, China).

### 2.3. Plasma and Urine Biochemical Analyses

The plasma and urine were thawed by incubation at 37°C for 15 min and centrifuged at 4,000 rpm for 5 min before use. The concentrations of creatinine in plasma (Scr) and in urine (Ucr) were measured by a Beckman Coulter AU5800 clinical chemistry analyzer (Beckman Coulter, Brea, CA, USA). The concentration of urinary albumin was measured by using a mouse albumin ELISA kit (Abcam, Cat: 1089792). The concentration of urinary NAG was measured by using an assay kit (Jiancheng, Nanjing, China).The creatinine clearance (Ccr) was determined by Scr and Ucr [31]. The urinary albumin/creatinine ratio (ACR) was determined by urinary albumin and Ucr [32].

### 2.4. Renal Histopathological Analysis

Renal tissues were fixed in 4% paraformaldehyde solution for 12 hours. Then, the renal tissues were embedded in paraffin, cut at 5 *μ*m, later deparaffinized in xylene and rehydrated through graded alcohols for histopathological examination. Glomerular morphology was examined by hematoxylin & eosin (H&E) staining. Renal glycogen accumulation was examined by periodic acid Schiff (PAS) staining. Renal fibrosis was examined by Masson's trichrome (MT) staining [12]. The tissue sections were then observed through a light microscope (Leica DMI3000B, Wetzlar, Germany). Three samples were selected randomly from each group, and 20 random fields were observed per sample.

### 2.5. Electron Transmission Microscopy

For electron transmission microscope evaluation of glomerular filtration barrier, the renal cortex was fixed in 2.5% glutaraldehyde solution and processed subsequently by a standard protocol, including dehydration, embedding, and sectioning as reported previously [33]. The renal cortex ultrastructure was observed through a Hitachi 7650 transmission electron microscope (Hitachi, Tokyo, Japan). Three samples were selected randomly from each group, and more than four random glomeruli fields were observed per sample. The GBM thickness was determined by a perpendicular line drawn from the endothelial to the epithelial edge (more than 6 photographs in each sample). The average podocyte foot process width (FPW) was determined by (1) (more than 4 photographs in each sample) [34]. Image analysis was performed using ImageJ software.

(1) $$\begin{array}{c} FPW=\frac{\pi }{4}\times \frac{\sum GBM\;\;length}{\sum foot\;\;process} \end{array}$$

### 2.6. Quantitative RT-PCR

Total RNA from the renal cortex was isolated using an RNAiso Plus (TaKaRa Bio Inc., Shiga, Japan). The reverse transcription (RT) reactions were performed with a PrimeScript™ RT reagent kit (TaKaRa Bio, Inc., Shiga, Japan) according to the manufacturer's instructions. Quantitative analysis of specific mRNA expression was performed with a Bio-Rad CFX96 Real-Time System (Bio-Rad Laboratories, USA). The sequences of the forward and reverse primers were as follows: 5′-CAACAATTCCTGGCGTTACCTTGG-3′ and 5′-GAAAGCCCTGTATTCCGTCTCCTT-3′ for the transforming growth factor *β*1 (TGF-*β*1) gene, 5′-CTCCACCCGAGTTACCAATGACAA-3′ and 5′-CCAGAAAGCTCAAACTTGACAGGC-3′ for the connective tissue growth factor (CTGF) gene, 5′-CACCCACGACAGAAGGAGA-3′ and 5′-ATGTCCACCAGGGTCTCAAT-3′ for the vascular endothelial growth factor (VEGF) gene, 5′-GGCCTGAACCAGCCTACAG-3′ and 5′-TGAGCTTAAAGCCAGCGTCA-3′ for the fibronectin 1 (FN1) gene, 5′-ATCTCCTGGTGCTGATGGAC-3′ and 5′-ACCTTGTTTGCCAGGTTCAC-3′ for the collagen I (Col I) gene, 5′-GCAACGGTACAAAGGGAGAGAG-3′ and 5′-CTTCATTCCTGGTAACCCTGGTG-3′ for the collagen IV (Col IV) gene, 5′-ATGCCATCCTGCGTCTGGAC-3′ and 5′-AGCATTTGCGGTGCACGATGG-3′ for the internal control *β*-actin gene. The thermocycling conditions were initiated at 95°C for 90 s, followed by 39 PCR cycles of denaturation at 95°C for 10 s, annealing at 60°C for 30 s, and extension at 72°C for 30 s.

### 2.7. Metabonomics Analysis

The plasma and urine were thawed by incubation at 37°C for 15 min and centrifuged at 4,000 rpm for 5 min before use. Urine (30 *μ*L) was added with an equal volume of 10 mg/mL urease (Sigma-Aldrich, St. Louis, MO) in normal saline, and the mixtures were then incubated at 37°C for 1 hour to remove the urea because the presence of high levels of urea would interfere with the results. Renal tissue was homogenized by a liquid nitrogen-chilled mortar and pestle. For plasma (50 *μ*L), urine (60 *μ*L in total), and renal tissue (20 mg), 200 *μ*L methanol, 250 *μ*L methanol, and 800 *μ*L methanol:H_2_O (80:20) were added to each bio-sample separately, which contained 5 *μ*g/mL internal standard A, [^13^C_2_]-myristic acid (Sigma-Aldrich, St. Louis, MO), for GC-MS and 15 *μ*g/mL internal standard B, 5-^13^C-glutamine (Cambridge Isotope Laboratories, Andover, MA, USA), for HPLC-QTOF/MS. Then, the mixtures were vigorously extracted for 5 min and placed at 4°C for 1 hour. Next, the mixtures were centrifuged at 2,0000 g for 10 min with the SORVALL BiofugeStratos centrifuge (Sollentum, Germany). An aliquot of 100 *μ*L supernatant was transferred to a GC or LC vial, which was then evaporated to dryness with a SPD2010-230 SpeedVac Concentrator (Thermo Savant, NY, USA). For GC/MS, the dried residue of plasma, urine, and renal tissue was dissolved in 30 *μ*L of 10 mg/mL methoxyamine (Sigma-Aldrich, St. Louis, MO) in pyridine, and the methoximation reaction was performed for 16 h at 22±2°C. Then, trimethylsilylation for 1 hour was carried out by 30 *μ*L of N-methyl-N (trimethylsilyl) trifluoroacetamide with 1% trimethylchlorosilane (Sigma-Aldrich, St. Louis, MO). Finally, the solution was vortexed again for 30 s after 30 *μ*L n-heptane was added, and 0.4 *μ*L was injected for GC-MS analysis. ForHPLC-QTOF/MS, the residue was redissolved with 100 *μ*L distilled water and centrifuged at 18,000 rpm for 5 min. Finally, 80 *μ*L supernatant was transferred to an LC vial, and 10 *μ*L was injected for HPLC-QTOF/MS analysis. In addition, 12 plasma (or urine, renal tissue) samples were selected randomly and mixed as quality control (QC) samples, and the QC samples were injected every ten samples to monitor the stability of the analysis.

The GC-MS analysis was performed as reported previously [11, 35]. Briefly, a Shimadzu GCMSQP2010 (Shimadzu Corp., Tokyo, Japan) was equipped with an RTx-5MS column (30 m × 0.25 mm inner diameter fused-silica capillary column chemically bonded with a 0.25 *μ*m cross bond, 5% diphenyl/95% dimethyl polysiloxane (Restek Corporation, PA, USA), for chromatographic separation). The temperature of the carrier gas (helium) was set with the following gradient: 0-3 min 80°C, 3-14 min 80-300°C, and 14-19 min 300°C. The injection temperature was set at 250°C, and the ion source temperature was set at 220°C. The eluate was introduced into the mass spectrometer through the transfer line, which was ionized with a current beam of 70 eV. The mass scan ranged between m/z 50–700, and the detector voltage was set at −1050 V. The metabolite identification was performed with the GC-MS Labsolutions software (Version 2.61, Shimadzu Corp., Tokyo, Japan) and databases such as Wiley 9 (Wiley-VCH Verlag GmbH & Co. KGaA, Weinheim, Germany), National Institute of Standards and Technology (NIST) library 2.0 (2012) and the in-house mass spectra library database established in our laboratory.

The HPLC-QTOF/MS analysis was carried out as previously reported in our laboratory [36]. The chromatographic separation of the analyses was achieved with an Amide XBridge HPLC column (3.5 *μ*m; 4.6 mm × 100 mm; Waters, USA). The column temperature was set to 40°C. The HPLC system consisted of a LC-30A binary pump, a SIL-30AC autosampler and a CTO-30AC column oven (Shimadzu, Japan) coupled with a hybrid quadrupole time-of-flight tandem mass spectrometer (AB SCIEX TripleTOF® 5600, Foster City, CA). The mobile phase was composed of 5 mM ammonium acetate in ultrapure water (regulating to pH=9 with ammonia) plus 5% acetonitrile (solvent A) and acetonitrile (solvent B). The mobile phase was delivered at 0.4 ml/min using a solvent gradient as follows: 0-3 min, 85% B; 3-6 min, 85-30% B; 6-15 min, 30-2% B; 15-18 min, 2% B; 18-19 min, 2-85% B; and 19-26 min, 85% B. A Turbo V electrospray ionization (ESI) was used in MS detection with negative ion modes. The ESI source conditions setting and metabolite identification were performed as previously reported. The automatic calibration was carried out every eight samples. PeakView and MultiQuant 2.0 analytical software from AB SCIEX were used for data analysis.

### 2.8. Metabolomic Data Processing

The metabolites detected by multimetabonomics analyses were relatively quantified. After normalization by the internal standard A (GC-MS) or B (HPLC-QTOF/MS), the peak areas were weighted followed by volume for urine and weight for kidney. To compare the difference in each group, the SIMCA-P 13 Software package (Umetrics, Umeå, Sweden) was used for multivariate statistical analysis. Principal components analysis (PCA), an unsupervised method, was used to explicate the overall distribution of all samples. Partial least squares discriminant analysis (PLS-DA), a supervised method, was used to confirm the general separation of groups. Orthogonal partial least squares discriminant analysis (OPLS-DA), an extension of PLS-DA, was used to distinguish between two groups and identify the differential metabolites. In addition, metabolomics pathway analysis based on differential metabolites was performed with MetaboAnalyst (http://www.metaboanalyst.ca), which is a website tool including the KEGG (http://www.genome.jp/kegg/) and HMDB (http://www.hmdb.ca/) databases.

### 2.9. Statistical Analysis

Statistical analyses were performed using SPSS software (version 19.0, IBM, USA). All results are expressed as the mean ± standard error of the mean (SEM). An unpaired Student's t-test was used to detect differences in two groups, and one-way ANOVA was used to detect differences in multiple groups. A p value of <  0.05 was considered statistically significant.

## 3. Results

### 3.1. Biochemical Parameters

The average body weight (g/mice) and ingestion (g/mice/day) in each group from 7 to 18 weeks are shown in Tables S1 and S2 in the Supplementary Material. The body weight and ingestion of db/db mice were both significantly greater than those of db/m mice, as expected. HS had no impact on the body weight and ingestion of db/db mice.

**Figure 1:**
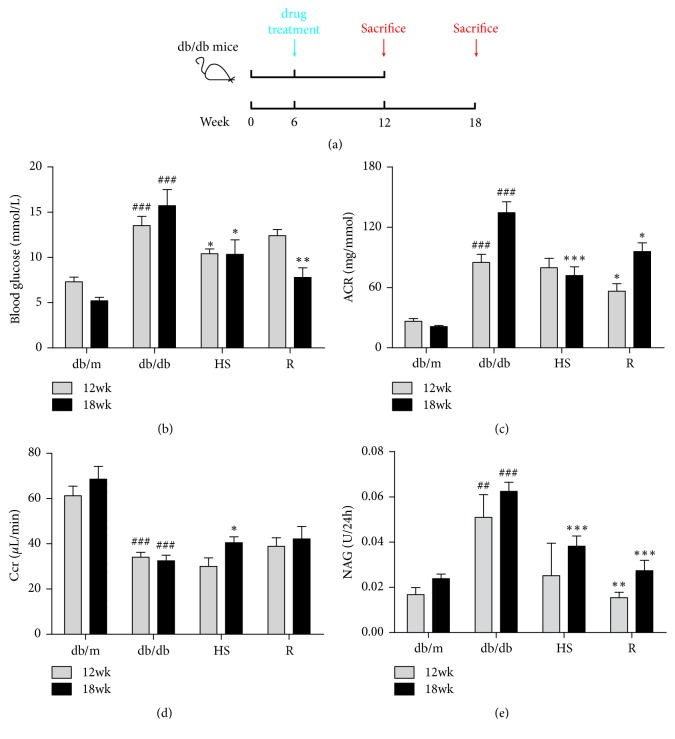
HS reduced fasting plasma glucose and improved renal function. (a) Schematic diagram showing the treatment regimen of male db/db mice. (b) Fasting plasma glucose. (c) Urinary albumin/creatinine ratio (ACR). (d) Creatinine clearance (Ccr). (e) Urinary N-acetyl-beta-D-glucosaminidase (NAG) level. The data are expressed as the mean ±  SEM. ^###^p < 0.001 versus the db/m group in each week age; ^*∗*^p < 0.05, ^*∗∗*^p < 0.01, and ^*∗∗∗*^p < 0.001 versus the db/db group in each week age.

Compared to the db/m group, 12- or 18-week-old db/db mice all showed an elevated fasting plasma glucose level (Figure 1(b)). Glomerular filtration dysfunction was characterized by an increasing ACR (Figure 1(c)) and a decreasing Ccr (Figure 1(d)) level. Renal tubular injury was characterized by elevated NAG (Figure 1(e)) level. Treatment with HS reduced fasting plasma glucose, urinary ACR, and NAG, while increasing the Ccr level in the db/db mice at 18 weeks of age.

### 3.2. Histopathological and Electron Micrographic Inspection of the Microstructure in the Renal Cortex

**Figure 2:**
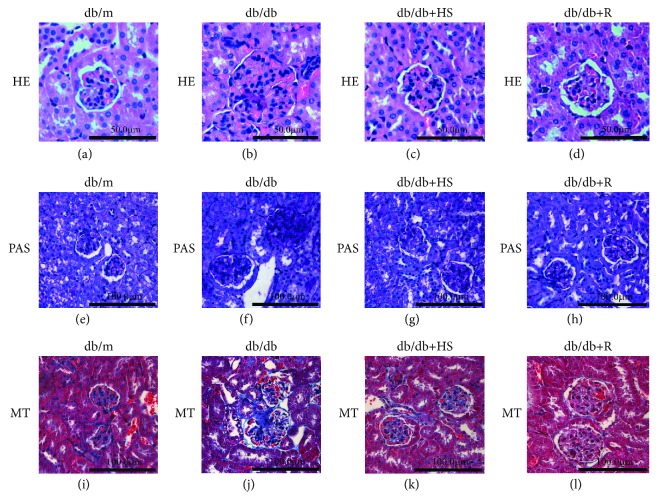
HS reduced glomeruli hypertrophy, renal glycogen accumulation, and fibrosis of db/db mice. (a-d) Hematoxylin & eosin (H&E) staining, ×400 magnification. (e-h) Periodic acid Schiff (PAS) staining, ×200 magnification. (i-l) Masson's trichrome (MT) staining, ×200 magnification.

Compared to the normal db/m control mice, 18-week-old db/db mice showed glomeruli hypertrophy (Figure 2(b)), renal glycogen accumulation (Figure 2(f)), and fibrosis (Figure 2(j)). Treatment with HS partly reestablished the normal morphology of the kidney (Figures 2(c), 2(g), and 2(k)).

**Figure 3:**
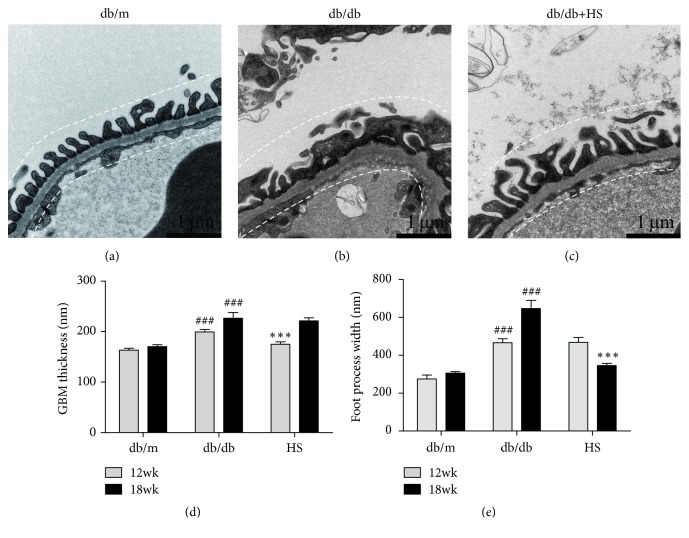
HS normalized the disordered ultrastructure of the glomerular filtration barrier in the diabetic kidney. (a-c) The renal field of transmission electron microscopy from mice at 18 weeks of age, ×20000 magnification. (d) GBM thickness. (e) The average podocyte foot process width (FPW). The data are expressed as the mean ±  SEM. ^###^p < 0.001 versus the db/m group in each week age. ^*∗∗∗*^p< 0.001 versus the db/db group in each week age.

Additionally, ultrastructural examination showed that the glomerular filtration barrier is an important indicator for DN [37]. Compared with the db/m group, db/db mice showed GBM thickening (Figures 3(b) and 3(d)) and increased podocyte foot process width (FPW) (Figures 3(b) and 3(e)), indicating that the glomerular filtration barrier was damaged during diabetic nephropathy. Treatment with HS for 6 weeks reduced the GBM thickness (Figure 3(d)), and treatment for 12 weeks distinctly reduced the FPW (Figure 3(e)).

### 3.3. RT-PCR Analysis of Fibrosis Factors in the Renal Cortex

**Figure 4:**
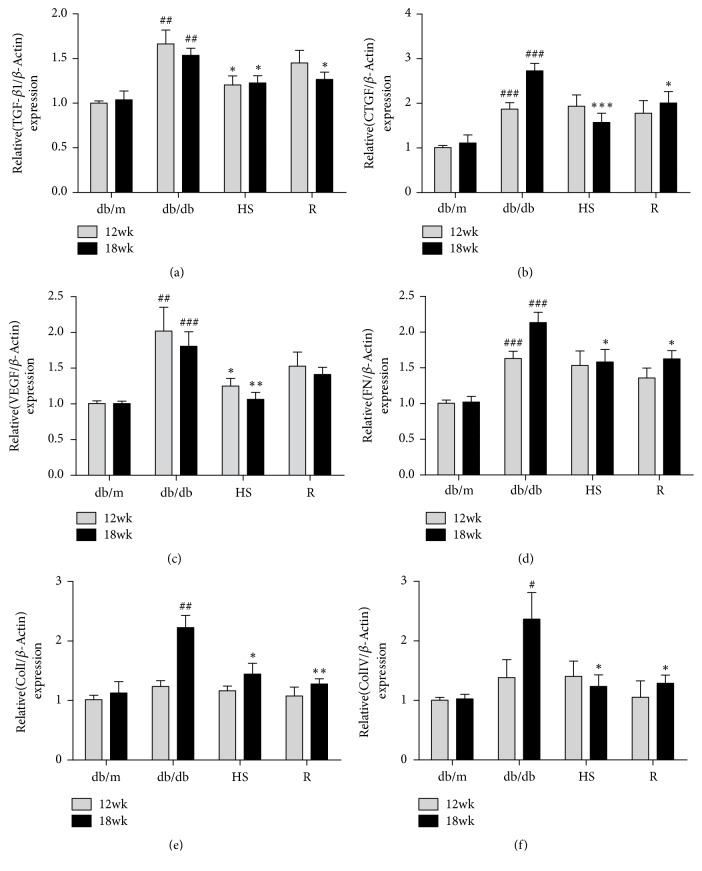
HS inhibited the gene expression of fibrosis-related factors in diabetic kidneys. (a) Transforming growth factor-*β*1 (TGF-*β*1). (b) Vascular endothelial growth factor (VEGF). (c) Connective tissue growth factor (CTGF). (d) Fibronectin (FN). (e) Collagen type I (Col I). (f) Collagen type IV (Col IV). The data are expressed as the mean  ± SEM. ^#^p < 0.05, ^##^p < 0.01, and ^###^p < 0.001 versus the db/m group in each week age; ^*∗*^p < 0.05, ^*∗∗*^p< 0.01, and ^*∗∗∗*^p < 0.001 versus the db/db group in each week age.

The renal fibrosis mentioned above is an important part of the pathological changes of DN [38], and TGF-*β* signaling plays a significant role in the process of fibrosis [39]. To confirm the pathologic process, we performed RT-PCR analysis of fibrosis factors in the renal cortex. Compared to the db/m group, the expression of TGF-*β*1 was significantly elevated in the renal cortex of db/db mice (Figure 4(a)), and the downstream fibrosis factors CTGF, VEGF, FN, Col I, and Col IV were also significantly increased (Figures 4(b)-4(f)). HS treatment for 12 weeks significantly reduced the gene expression of these fibrosis factors.

### 3.4. HPLC-QTOF/MS and GC-MS Data Analysis of Plasma, Urine, and Kidney

**Figure 5:**
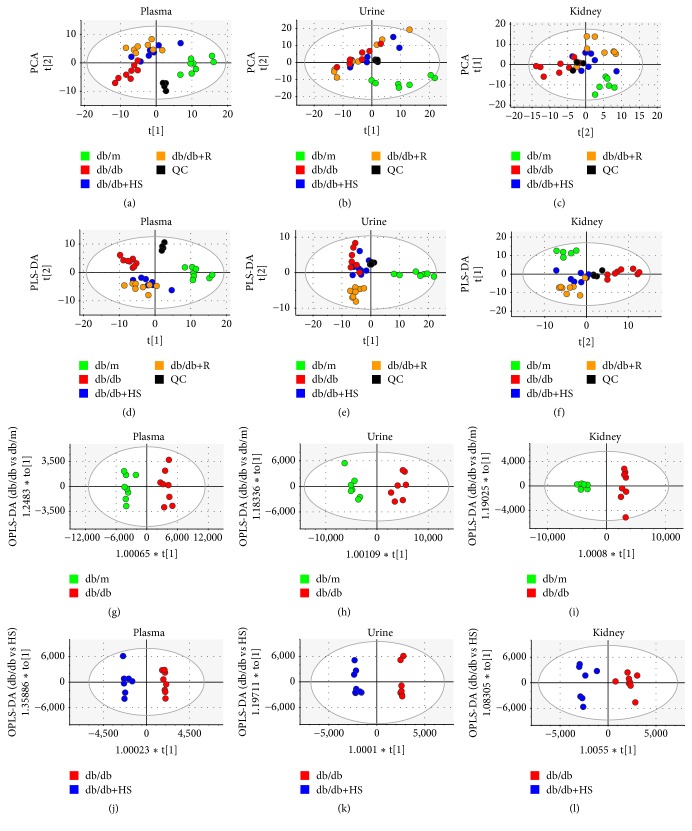
Metabolic patterns of mice at 18 weeks based on multivariate statistical analysis of data from the GC-MS and HPLC-QTOF/MS showed that HS regulated the metabolic disorder of diabetic mice towards normal levels. Score plots of the four groups and their PCA models for plasma (a), urine (b), and kidney (c) data. The PLS-DA models for plasma (d), urine (e), and kidney (f) data. Db/db versus db/m groups and their OPLS-DA models for plasma (g), urine (h), and kidney (i) data. Db/db versus HS groups and their OPLS-DA models for plasma (j), urine (k), and kidney (l) data. The blank dots represent the QC group. The green dots represent the db/m group. The red dots represent the db/db group. The blue dots represent the HS treatment group. The brown dots represent the positive treatment group.

With a multimetabolomics platform of GC-MS and HPLC-QTOF/MS, the metabolites in the plasma, urine, and kidneys of 18-week-old mice were analyzed to investigate metabolic changes in the diabetic state and the regulation of HS. After the data processing mentioned in the methods section, a total of 227 compounds were identified in plasma, 275 compounds in urine and 265 compounds in kidney. Typical GC-MS and UPLC-QTOF/MS spectra obtained for the plasma, urine, and renal cortex are shown in Figures S1 and S2 in the Supplementary Material. The PCA, PLS-DA, and OPLS-DA models were calculated, and the associated model parameters are shown in Table 1. Both unsupervised PCA and supervised PLS-DA score plots showed that the quality control (QC) samples clustered together tightly in plasma (Figures 5(a) and 5(d)), urine (Figures 5(b) and 5(e)), and kidney (Figures 5(c) and 5(f)) samples, which suggested a stable platform and reliable data. The db/m group and db/db group at the 18th week separated clearly in three types of bio-samples, suggesting the presence of metabolic differences in the plasma, urine, and kidney during diabetes mellitus. Based on the data from the plasma and kidney samples, the HS group moved closely to the db/m group. Based on the urinary data, the HS group moved slightly to the db/m group. These findings showed that HS regulated the perturbed metabolism of diabetic mice towards normal levels.

### 3.5. Perturbed Metabolites and Metabolic Pathways Involved in Plasma, Urine, and Kidney

**Figure 6:**
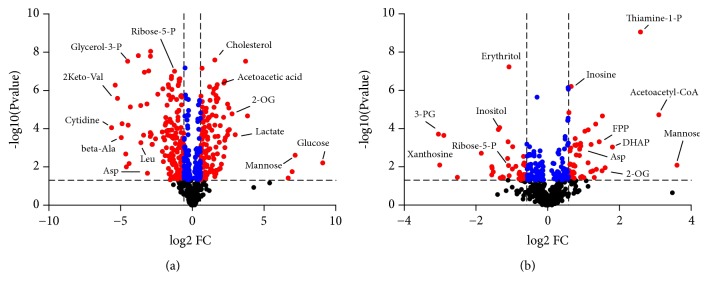
The metabolite volcano plots of the db/m group versus db/db group (a) and the db/db group versus HS treatment group (b) in three biosamples; the red dots represent the metabolites with a p value>0.05 and fold change>1.5 or <0.67, the blue dots represent the metabolites with a p value>0.05 and 0.67<fold change<1.5, and the blank dots represent the nonsignificant metabolites.

The OPLS-DA model was established to differentiate groups and identify the discriminant metabolites in the db/db group versus the db/m group (Figures 5(g)–5(i)) and the db/db group versus the db/db+HS group (Figures 5(j)–5(l)). The R2Y values over 0.9 and the Q2 values over 0.8 (Table 1) suggested the good fitness and high predictability of each model. Moreover, there were large differences between the db/db group and the db/m group (Figure 6(a)). The HS treatment significantly changed the metabolites of db/db mice (Figure 6(b)).

**Figure 7:**
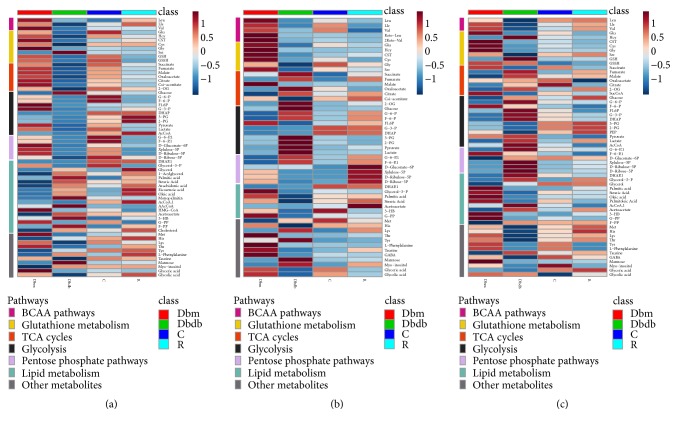
Heatmap exhibiting the intensities of differential metabolites in the plasma (a), urine (b), and kidney (c). Pathways included BCAA metabolism, glutathione metabolism, TCA cycles, glycolysis, and the pentose phosphate pathway.

**Figure 8:**
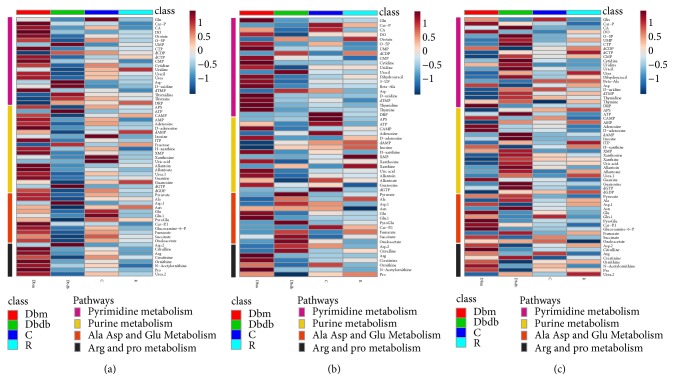
Heatmap exhibiting the intensities of differential metabolites in the plasma (a), urine (b), and kidney (c). Pathways included pyrimidine metabolism, purine metabolism, Ala, Asp, and Glu metabolism and Arg and Pro metabolism.

**Figure 9:**
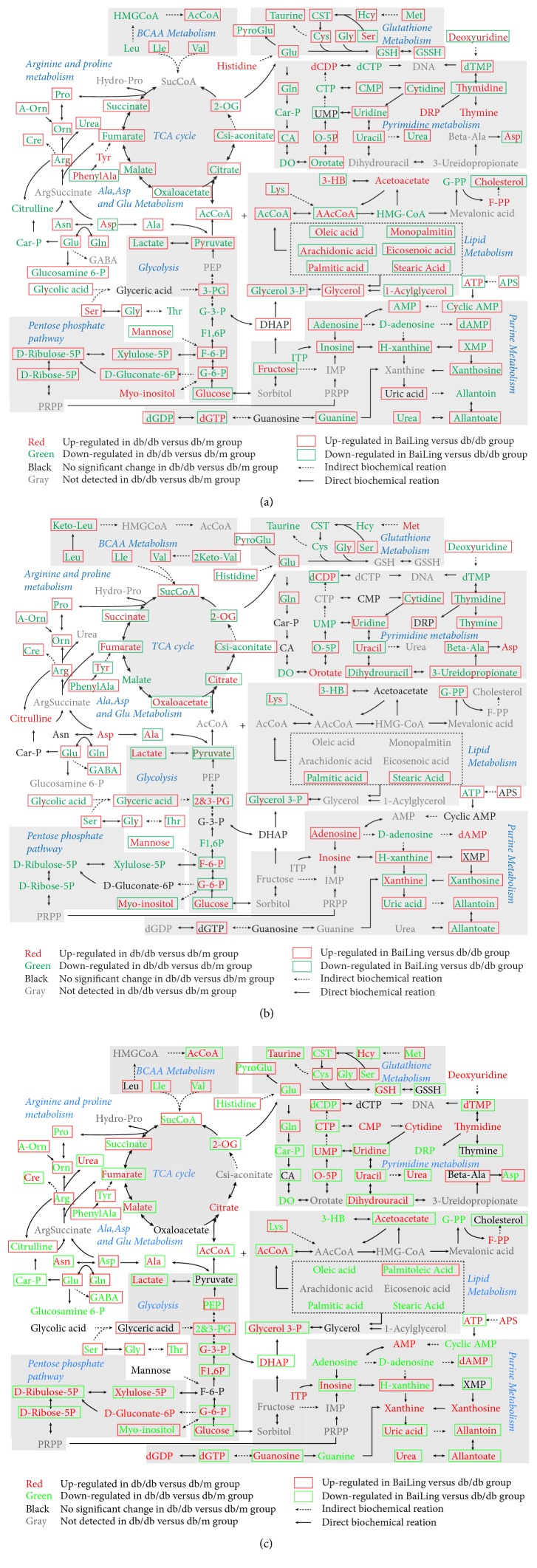
The differential metabolites and associated metabolic pathways involved in db/db mice and the treatment with HS in plasma (a), urine (b), and kidney (c).

The differential metabolites with statistical significance (p value <  0.05) in the plasma, urine, and kidney were selected from the db/db group versus db/m group and the db/db group versus HS treatment group at 12 and 18 weeks of age. Based on the KEGG and HMDB databases, pathway analysis showed substantial metabolic disturbance in the db/db mice, including the tricarboxylic acid cycle (TCA), glycolysis, pentose phosphate pathway, lipid metabolism, Arg and Pro metabolism, Ala, Asp, and Glu metabolism, branched amino acid (BCAA) metabolism, glutathione metabolism, pyrimidine metabolism, purine metabolism, and others (Figures 7, 8, and 9). The abbreviations for metabolites involved in these pathways are shown in Table S3 in the Supplementary Material. Heatmaps were generated for each bio-sample to exhibit the intensities of differential metabolites in different groups (Figures 7 and 8). In detail, the Lle and Val involved in the BCAA pathway; the Glu, Gln, CST, Cys, and Gly involved in glutathione metabolism; and the Pro, A-Orn, Orn, and Arg involved in Arg and Pro metabolism were all significantly reduced in the plasma, urine, and kidney of db/db mice, which indicated an imbalance of the biosynthesis of proteins and the turnover of amino acids in DN mice. The HS treatment ameliorated the deficiency of the amino acids in all three types of bio-samples, including Lle, Val, Glu, Gln, Gly, Pro, A-Orn, Orn, and Arg. In contrast to the elevation of glucose in the three types of bio-samples, there was an overall decrease in the metabolites involved in the TCA cycle, glycolysis, and the pentose phosphate pathway in the plasma of db/db mice, such as the succinate, 2-OG, cis-aconitate, citrate, oxaloacetate, malate, fumarate, AcCoA, pyruvate, lactate, 3-PG, G-3-P, F1, 6P, F-6-P, G-6-P, D-Ribulose-5P, D-Ribose-5P, D-Gluconate-6P, and xylulose-5P. In contrast, there was an increase in the metabolites involved in lipid metabolism in the plasma of db/db mice, such as 1-acylglycerol, glycerol, palmitic acid, arachidonic acid, oleic acid, monopalmitin, eicosenoic acid, stearic acid, AAcCoA, acetoacetate, 3-HB, F-PP, and cholesterol, indicating an imbalance of glycolipid metabolism during diabetes mellitus. HS partially regulated the disordered metabolites of the TCA cycle, glycolysis, pentose phosphate pathway, and lipid metabolism towards normal levels in the plasma, including succinate, 2-OG, cis-aconitate, citrate, oxaloacetate, malate, fumarate, AcCoA, pyruvate, lactate, 3-PG, F-6-P, G-6-P, D-Ribulose-5P, D-Ribose-5P, D-Gluconate-6P, xylulose-5P, 1-acylglycerol, palmitic acid, arachidonic acid, oleic acid, monopalmitin, eicosenoic acid, stearic acid, AAcCoA, 3-HB, and cholesterol.

Interestingly, in urine, the levels of most intermediates in the TCA cycle and glycolysis showed opposite trend to that in plasma, such as the 2-OG, citrate, oxaloacetate, fumarate, and succinate involved in the TCA cycle and the pyruvate, lactate, 2-PG, 3-PG, F-6-P, and G-6-P involved in glycolysis. The HS treatment rebalanced the disordered metabolites in urine, including 2-OG, citrate, oxaloacetate, fumarate, succinate, pyruvate, lactate, 2-PG, 3-PG, F-6-P, and G-6-P.

Based on the metabolomics data from the kidneys of db/db mice, we observed increasing levels of the primary metabolites involved in the TCA cycle, glycolysis, and the pentose phosphate pathway, such as 2-OG, citrate, malate, fumarate, AcCoA, lactate, G-3-P, F1,6P, G-6-P, D-Ribulose-5P, D-Ribose-5P, D-Gluconate-6P, and xylulose-5P, indicating an upregulated energy metabolism. In addition, the deoxyuridine, dTMP, thymidine, CTP, CMP, cytidine, uridine, UMP, O-5P, uracil, dihydrouracil, and urea involved in pyrimidine metabolism and the APS, ATP, AMP, dAMP, ITP, inosine, xanthosine, xanthine, guanosine, dGTP, dGDP, uric acid, allantoin, allantoate, and urea involved in purine metabolism were significantly increased in the kidneys of db/db mice, indicating that nucleic acid synthesis was also active. The upregulated energy metabolism and biosynthesis of nucleic acids indicated an overloaded kidney of the db/db mice. The HS treatment regulated the metabolites of the TCA cycle, glycolysis, pentose phosphate pathway, pyrimidine metabolism, and purine metabolism towards normal levels, including 2-OG, malate, fumarate, AcCoA, lactate, G-3-P, F1, 6P, G-6-P, D-Ribulose-5P, D-Ribose-5P, xylulose-5P, dTMP, CTP, uridine, UMP, O-5P, uracil, dihydrouracil, urea, ATP, dAMP, inosine, guanosine, dGTP, uric acid, allantoin, and allantoate.

## 4. Discussion

The main reason for the controversy over the pharmacological effect of HS is the lack of knowledge of the active constituents. Some constituents have been reported in HS, such as cordycepin (3′-deoxyadenosine), cordycepic acid (d-mannitol), polysaccharides, and amino acids [25, 26]. However, the pharmacological effects of these constituents have been considered limited for a long time. For example, cordycepic acid (d-mannitol) is an excipient for chewable tablets [40]. Recent research has indicated that HS has antihepatoma activity [41]. The polysaccharides isolated from HS also play an important role in antiobesity, anti-inflammation, and insulin resistance [42]. Therefore, the pharmacological effect of HS has been gradually recognized. The renal protection of HS has been reported [22, 27], but its effect on DN is still controversial today. In this study, the db/db mice showed a remarkable accumulation of blood glucose in terms of time, indicating the occurrence of diabetes mellitus. We used this well-established model to investigate the effects of HS on renal injury induced by diabetes (Figure 1(a)). HS reduced FBG, urinary ACR, and NAG accumulation and increased the Ccr level. The classic renal pathological observations also indicated that HS decreased glomeruli hypertrophy, renal glycogen accumulation, and fibrosis and ameliorated glomerular filtration barrier damage. Meanwhile, the RT-PCR results showed elevated gene expression of fibrosis-related TGF-*β*1 and downstream factors in the renal cortex of db/db mice, and HS significantly reduced the expression of these factors. These results suggested that HS could protect against renal injury in db/db mice.

Metabonomics is an important means to explore the pharmacological mechanism of TCM with complex components. Therefore, a metabonomics analysis was utilized to explore the metabolism regulation of HS. The alterations of metabolites in plasma, urine, and kidney can reflect the systemic and renal metabolic disorders of db/db mice [43]. Previous studies have demonstrated that the levels of TCA intermediates in the serum of db/db mice showed an elevation at 6 weeks of age but a sharp drop from 8 weeks of age, which indicates that the utilization of glucose via TCA cycles could be inefficient during type 2 diabetes [44]. In addition, elevated levels of intermediates during TCA cycles (fumarate, 2-OG and citrate) and glycolysis (lactate) in the urine and kidney of db/db mice after 8 weeks have also been reported [10, 45, 46]. In our study, reduced levels of intermediates during TCA cycles and glycolysis occurred in the plasma of db/db mice at later stages. However, these related intermediates were elevated in urine, indicating the dysfunction of the kidney, which caused abnormal loss of these energy substrates from plasma to urine. In normal conditions, the energy substrates coupled with Na^+^ are cotransported from the lumen into the proximal tubular cells [47]. Combined with the renal glomerular and tubular damage during DN mentioned above, impaired reabsorption of these intermediates by renal tubules may be a potential cause. The results showed that HS could restore the reduced or elevated intermediate levels during TCA cycles and glycolysis in plasma or urine, which also indicated the protective effects of HS on renal dysfunction.

For the metabolic analysis of the kidney, the elevated levels of intermediates during TCA cycles, glycolysis, and the pentose phosphate pathway indicated the enhanced energy metabolism in the diabetic kidney. Meanwhile, the elevated levels of intermediates during pyrimidine metabolism and purine metabolism indicated the enhanced nucleotide metabolism. The excessively activated energy metabolism and nucleotide metabolism may be a response to ‘overload work' of the kidney and further impair renal function during diabetes mellitus. The results showed that HS regulated the activated energy and nucleotide metabolism to normal levels in the kidney, illustrating that HS could restore renal metabolic disorders during diabetes mellitus.

Another interesting phenomenon occurred in plasma in which elevated levels of intermediates in lipid metabolism were found in db/db mice, which is opposite to glucose metabolism (TCA cycle, glycolysis, and pentose phosphate pathway) and indicates the disordered glycolipid metabolism during diabetes mellitus. In addition, the reduced levels of amino acids in BCAA metabolism, Glutathione metabolism, Arg and Pro metabolism, and Ala, Asp, and Glu metabolism occurred simultaneously in plasma, urine, and kidney. Amino acids are used as substitutes when the energy substrates in glucose metabolism are insufficient [48, 49]. Because of the abnormal loss of energy substrates from plasma to urine mentioned above, the body may consume amino acids to fight against hunger. HS also played a role in the regulation of glycolipid metabolic disorders and amino acid supplementation in the whole system.

Clinical patients with diabetic nephropathy usually show significant glomerular filtration barrier damage [50, 51] and metabolic profile changes [52]. In our study, we demonstrated that HS preserved renal injury and modulated metabolic disorder in db/db mice, suggesting that HS could play an important role in clinical practice for DN. However, some limitations were present in the current research. First, we selected a single effective dose from the result of preliminary experiments instead of multiple doses because of the cost of the db/db mice. Secondly, in consideration of the level of urinary NAG and the contrary changes in metabolites in the plasma and urine of db/db mice, the underlying mechanisms of HS for renal tubular reabsorption warrant further investigation. Third, owing to the complex composition of HS, it was impossible to obtain each single component for study. We will study the effects of different ethanol extracts from HS on DN in future studies.

## 5. Conclusions

In summary, the renal protection of HS was observed in db/db mice, as shown by the inhibition of renal glycogen accumulation, fibrosis, and proteinuria. These protective effects might be mediated, at least in part, by the reduced blood glucose, blocked TGF-*β* signaling, and normalized ultrastructure of the glomerular filtration barrier. In addition, a multimetabonomic platform combined with GC-MS and HPLC-QTOF/MS was first applied in this study to reveal that HS ameliorated glycolipid metabolic disorders and deficiency of amino acids in the whole system and regulated the excessive activated energy and nucleotide metabolism to normal levels in the diabetic kidney. The obvious regulation of the metabolic disorders further explains the renal protection of HS. These results provide a reference for resource utilization and the further development of HS.

## Figures and Tables

**Table 1:** Parameters associated with each model as referred to in Figure 5.

Model number	Type	Components	R2X (cum)	R2Y (cum)	Q2 (cum)
a	PCA-X	6	0.632		0.334
b	PCA-X	4	0.677		0.484
c	PCA-X	6	0.640		0.331
d	PLS-DA	6	0.617	0.941	0.804
e	PLS-DA	2	0.338	0.437	0.244
f	PLS-DA	8	0.695	0.949	0.748
g	OPLS-DA	1+1+0	0.693	0.968	0.917
h	OPLS-DA	1+1+0	0.599	0.957	0.924
i	OPLS-DA	1+1+0	0.557	0.982	0.942
j	OPLS-DA	1+4+0	0.761	0.986	0.917
k	OPLS-DA	1+6+0	0.873	0.992	0.841
l	OPLS-DA	1+1+0	0.513	0.936	0.84

## Data Availability

The data used to support the findings of this study are available from the corresponding author upon request.

## Supplementary Materials

Supplementary Materials Table S1: the mice average body weight (g) (n=8) in each group from 6 to 18 week. Table S2: the mice average ingestion (g/mice/day) (n=8) in each group from 7 to 18 week. Table S3: the abbreviations for metabolites involved in metabolic pathways. Figure S1: representative GC-MS spectra of mice plasma (a), urine (b), and renal cortex (c). Figure S2: representative HPLC-QTOF/MS spectra of mice plasma (a), urine (b), and renal cortex (c).
