# Supplementary material for: Hirsutella sinensis Treatment Shows Protective Effects on Renal Injury and Metabolic Modulation in db/db Mice
Source: Evid Based Complement Alternat Med. 2019 Apr 4;2019:4732858. doi: 10.1155/2019/4732858 (PMC6475559; doi:10.1155/2019/4732858)
Supplement: Supplementary Materials — Table S1: the mice average body weight (g) (n=8) in each group from 6 to 18 week. Table S2: the mice average ingestion (g/mice/day) (n=8) in each group from 7 to 18 week. Table S3: the abbreviations for metabolites involved in metabolic pathways. Figure S1: representative GC-MS spectra of mice plasma (a), urine (b), and renal cortex (c). Figure S2: representative HPLC-QTOF/MS spectra of mice plasma (a), urine (b), and renal cortex (c). [file 4732858.f1.doc]

# Hirsutella Sinensis Treatment shows protective effects on Renal injury and Metabolic modulation in Db/db Mice

Zhenyao Lu,^1^ Sijia Li,^1^ Runbin Sun,^1^ Xue Jia,^1^ Chen Xu,^1^ Jiye Aa,^1*^and Guangji Wang^1*^

1 Jiangsu Provincial Key Laboratory of Drug Metabolism and Pharmacokinetics, China Pharmaceutical University, Nanjing 210009, China.

Correspondence should be addressed to Guangji Wang; [guangjiwang@hotmail.com](mailto:guangjiwang@hotmail.com); Jiye Aa; jiyea@cpu.edu.cn

## Supplementary Materials

Table S1: The mice average body weight (g) (n=8) in each group from 6 to 18 week.

| Week | db/m(g) | db/db(g) | db/db+C(g) | db/db+R(g) |
| --- | --- | --- | --- | --- |
| 6 | 23.54±1.36 | 34.89±2.95^###^ | 33.98±1.57 | 33.63±3.37 |
| 7 | 23.70±1.22 | 35.78±3.23^###^ | 35.62±2.11 | 34.12±3.96 |
| 8 | 26.99±1.11 | 40.46±2.96^###^ | 40.37±2.13 | 38.25±3.69 |
| 9 | 27.99±1.41 | 43.86±1.73^###^ | 44.49±2.56 | 41.88±2.75 |
| 10 | 28.52±1.45 | 45.29±2.83^###^ | 45.29±2.65 | 43.88±2.85 |
| 11 | 29.34±1.40 | 47.97±2.98^###^ | 46.36±3.04 | 46.42±3.07 |
| 12 | 30.43±1.38 | 50.66±2.59^###^ | 49.20±2.68 | 48.43±3.36 |
| 13 | 31.35±1.30 | 52.02±3.49^###^ | 51.46±2.59 | 50.45±3.56 |
| 14 | 32.17±1.35 | 53.98±3.70^###^ | 53.00±2.47 | 51.53±4.05 |
| 15 | 32.35±1.48 | 55.26±3.55^###^ | 54.20±2.40 | 53.26±3.53 |
| 16 | 33.31±1.64 | 57.22±2.98^###^ | 55.52±2.50 | 54.97±3.86 |
| 17 | 33.52±1.84 | 57.36±2.89^###^ | 55.87±2.73 | 55.53±3.95 |
| 18 | 32.85±2.57 | 56.49±4.48^###^ | 55.34±2.86 | 54.16±4.09 |

Compared with db/m mice, the db/db mice had an increasing body weight. Hirsutella sinensis (HS) or rhein had no significant change for body weight of db/db mice. The data are expressed as the mean ± SD. ###p < 0.001 vs. db/m group in each week.

Table S2: The mice average ingestion (g/mice/day) in each group from 7 to 18 week.

| Week | db/m  (g/mice/day) | db/db  (g/mice/day) | db/db+C  (g/mice/day) | db/db+R  (g/mice/day) |
| --- | --- | --- | --- | --- |
| 7 | 3.7 | 4.4 | 5.2 | 4.1 |
| 8 | 4.5 | 6.4 | 7.1 | 6.1 |
| 9 | 3.8 | 7.2 | 6.6 | 6.3 |
| 10 | 4.1 | 5.6 | 6.6 | 6.7 |
| 11 | 4.0 | 6.6 | 5.4 | 6.6 |
| 12 | 4.1 | 6.1 | 6.1 | 5.9 |
| 13 | 3.9 | 5.7 | 5.4 | 5.6 |
| 14 | 4.4 | 6.3 | 6.3 | 5.5 |
| 15 | 5.1 | 6.6 | 6.6 | 5.8 |
| 16 | 6.3 | 7.2 | 6.9 | 6.0 |
| 17 | 6.7 | 7.8 | 6.7 | 5.3 |
| 18 | 6.2 | 7.2 | 7.1 | 6.1 |
| average | 4.73±1.06 | 6.42±0.92^###^ | 6.35±0.67 | 5.83±0.68 |

Compared with db/m mice, the db/db mice had an increasing ingestion. Hirsutella sinensis (HS) or rhein had no significant change for ingestion of db/db mice. The data are expressd as the mean ± SD. ^###^p < 0.001 vs. db/m group.

Table S3: The abbreviations for metabolites involved in metabolic pathways.

| Abbreviation | Name | KEGG ID |
| --- | --- | --- |
| 1-Acylglycerol | 1-Acylglycerol | C01885 |
| dCDP | 2'-Deoxycytidine 5'-Diphosphate | C00705 |
| dCTP | 2'-Deoxycytidine 5'-Triphosphate | C00458 |
| dGDP | 2'-Deoxyguanosine 5'-Diphosphate | C00361 |
| dGTP | 2'-Deoxyguanosine 5'-Triphosphate | C00286 |
| 2-PG | 2-Phospho-D-Glycerate | C00631 |
| HMGCoA | 3-Hydroxy-3-Methylglutaryl-Coa | C00356 |
| 3-HB | 3-Hydroxybutyric Acid | C01089 |
| 3-PG | 3-Phosphoglycerate | C00197 |
| DO | 4,5-Dihydroorotic Acid | C00337 |
| Keto-Leu | 4-Methyl-2-Oxopentanoate | C00233 |
| PRPP | 5-Phosphoribosyl Diphosphate | C00119 |
| dTMP | 5-Thymidylic Acid | C00364 |
| D-Gluconate-6P | 6-Phosphogluconic Acid | C00345 |
| Acetoacetate | Acetoacetic Acid | C00164 |
| AAcCoA | Acetoacetyl-Coa | C00332 |
| AcCoA | Acetyl-Coa | C00024 |
| Adenosine | Adenosine | C00212 |
| AMP | Adenosine Monophosphate | C00020 |
| APS | Adenosine Phosphosulfate | C00224 |
| ATP | Adenosine Triphosphate | C00002 |
| Allantoate | Allantoic Acid | C00499 |
| Allantoin | Allantoin | C01551 |
| 2Keto-Val | Alpha-Ketoisovaleric Acid | C00141 |
| Arachidonic acid | Arachidonic Acid | C00219 |
| Beta-Ala | Beta-Alanine | C00099 |
| F1,6P | Beta-D-Fructose 1,6-Bisphosphate | C05378 |
| F-6-P | Beta-D-Fructose 6-Phosphate | C05345 |
| G-6-P | Beta-D-Glucose 6-Phosphate | C01172 |
| Car-P | Carbamoylphosphate | C00169 |
| Cholesterol | Cholesterol | C00187 |
| Csi-aconitate | Cis-Aconitic Acid | C00417 |
| Citrate | Citric Acid | C00158 |
| Citrulline | Citrulline | C00327 |
| Cre | Creatinine | C00791 |
| Cyclic AMP | Cyclic Adenosine Monophosphate | C00575 |
| Cytidine | Cytidine | C00475 |
| CMP | Cytidine Monophosphate | C00055 |
| CTP | Cytidine Triphosphate | C00063 |
| D-adenosine | Deoxyadenosine | C00559 |
| dAMP | Deoxyadenosine Monophosphate | C00360 |
| DRP | Deoxyribose 1-Phosphate | C00672 |
| Deoxyuridine | Deoxyuridine | C00526 |
| Fructose | D-Fructose | C02336 |
| Glc | D-Glucose | C00221 |
| Dihydrouracil | Dihydrouracil | C00429 |
| DHAP | Dihydroxyacetone Phosphate | C00111 |
| Mannose | D-Mannose | C00159 |
| D-Ribose-5P | D-Ribose 5-Phosphate | C00117 |
| D-Ribulose-5P | D-Ribulose 5-Phosphate | C00199 |
| Eicosenoic acid | Eicosenoic Acid | C16526 |
| F-PP | Farnesyl Diphosphate | C00448 |
| Fumarate | Fumaric Acid | C00122 |
| GABA | Gamma-Aminobutyric Acid | C00334 |
| G-PP | Geranyl Diphosphate | C00341 |
| Glucosamine 6-P | Glucosamine 6-Phosphate | C00352 |
| G-3-P | Glyceraldehyde 3-Phosphate | C00118 |
| Glyceric acid | Glyceric Acid | C00258 |
| Glycerol | Glycerol | C00116 |
| Glycerol 3-P | Glycerol 3-Phosphate | C00093 |
| Gly | Glycine | C00037 |
| Glycolic acid | Glycolic Acid | C00160 |
| Guanine | Guanine | C00242 |
| Guanosine | Guanosine | C00387 |
| Hydro-Pro | Hydroxyproline | C01157 |
| H-xanthine | Hypoxanthine | C00262 |
| Inosine | Inosine | C00294 |
| IMP | Inosine 5'-Monophosphate | C00130 |
| ITP | Inosine 5'-Triphosphate | C00081 |
| Ala | L-Alanine | C00041 |
| Arg | L-Arginine | C00062 |
| ArgSuccinate | L-Arginosuccinic Acid | C03406 |
| Asn | L-Asparagine | C00152 |
| Asp | L-Aspartic Acid | C00049 |
| CST | L-Cystathionine | C02291 |
| Cys | L-Cysteine | C00097 |
| Glu | L-Glutamic Acid | C00025 |
| Gln | L-Glutamine | C00064 |
| Histidine | L-Histidine | C00135 |
| Hcy | L-Homocysteine | C00155 |
| Lle | L-Isoleucine | C00407 |
| Lactate | L-Lactic Acid | C00186 |
| Leu | L-Leucine | C00123 |
| Lys | L-Lysine | C00047 |
| Malate | L-Malic Acid | C00149 |
| Met | L-Methionine | C00073 |
| PhenylAla | L-Phenylalanine | C00079 |
| Pro | L-Proline | C00148 |
| Ser | L-Serine | C00065 |
| Thr | L-Threonine | C00188 |
| Tyr | L-Tyrosine | C00082 |
| Val | L-Valine | C00183 |
| Mevalonic acid | Mevalonic Acid | C00418 |
| Monopalmitin | Monopalmitin | - |
| Myo-inositol | Myo-Inositol | C00137 |
| A-Orn | N-Acetylornithine | C00437 |
| 3-Ureidopropionate | N-Carbamoyl-Beta-Alanine | C02642 |
| Oleic acid | Oleic Acid | C00712 |
| Orn | Ornithine | C00077 |
| Orotate | Orotic Acid | C00295 |
| O-5P | Orotidylic Acid | C01103 |
| Oxaloacetate | Oxalacetic Acid | C00036 |
| GSSH | Oxidized Glutathione | C00127 |
| 2-OG | Oxoglutaric Acid | C00026 |
| Palmitic acid | Palmitic Acid | C00249 |
| Palmitoleic Acid | Palmitoleic Acid | C08362 |
| PEP | Phosphoenolpyruvic Acid | C00074 |
| PyroGlu | Pyroglutamic Acid | C01879 |
| Pyruvate | Pyruvic Acid | C00022 |
| GSH | Reduced Glutathione | C00051 |
| Sorbitol | Sorbitol | C00794 |
| Stearic Acid | Stearic Acid | C01530 |
| Succinate | Succinic Acid | C00042 |
| SucCoA | Succinyl-Coa | C00091 |
| Taurine | Taurine | C00245 |
| Thymidine | Thymidine | C00214 |
| Thymine | Thymine | C00178 |
| Uracil | Uracil | C00106 |
| Urea | Urea | C00086 |
| CA | Ureidosuccinic Acid | C00438 |
| Uric acid | Uric Acid | C00366 |
| Uridine | Uridine | C00299 |
| UMP | Uridine 5'-Monophosphate | C00105 |
| Xanthine | Xanthine | C00385 |
| Xanthosine | Xanthosine | C01762 |
| XMP | Xanthylic Acid | C00655 |
| Xylulose-5P | Xylulose 5-Phosphate | C00231 |


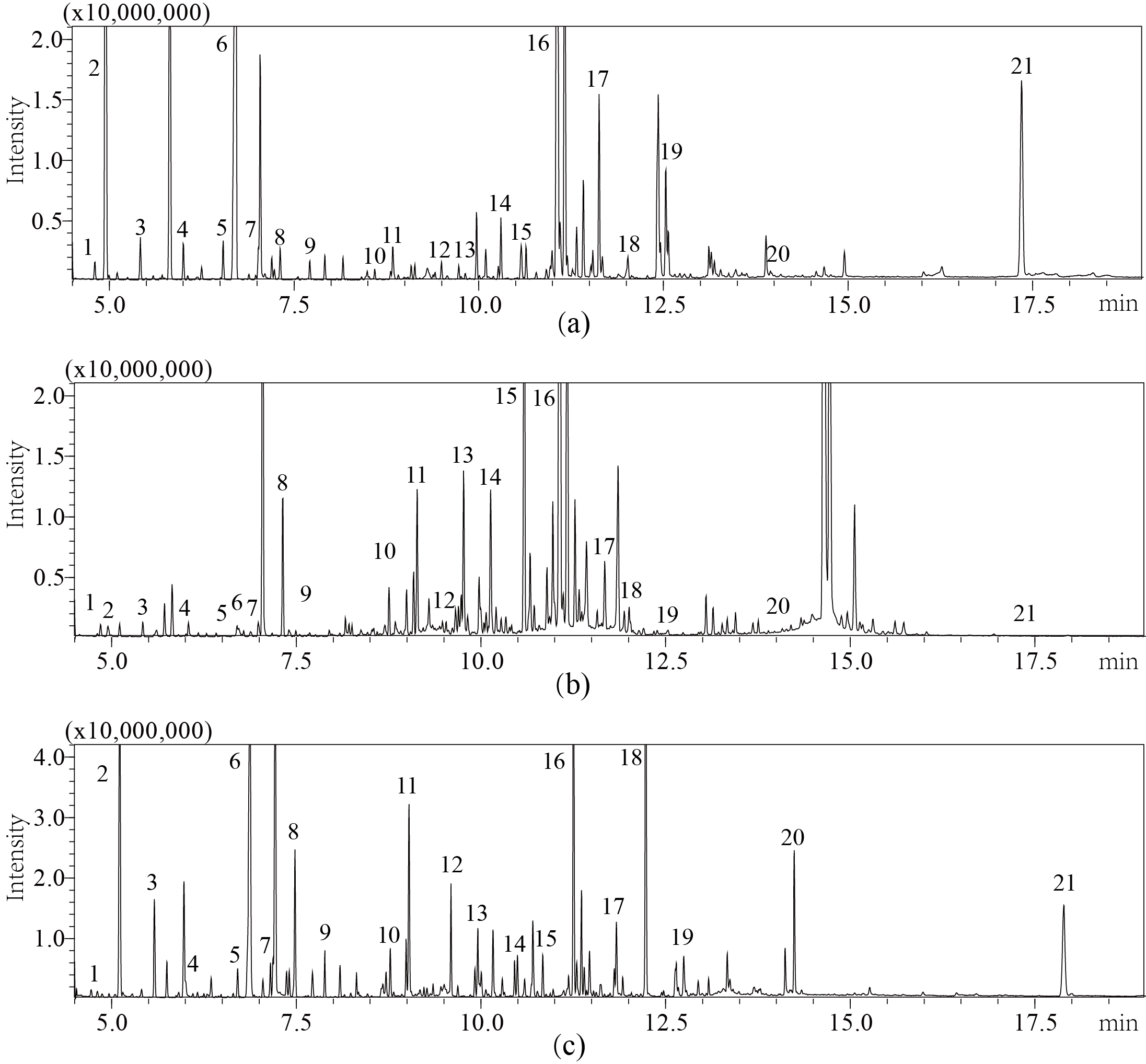


Figure S1: Representative GC-MS spectra of mice plasma (a), urine (b), and renal cortex (c). Typical compounds are identified and numbered as follows: 1, Pyruvate; 2, Lactate; 3, Alanine; 4, 3-Hydroxybutyrate; 5, Valine; 6, Urea; 7, Leucine; 8, Succinate; 9, Serine; 10, Malate; 11, Pyroglutamate; 12, Glutamate; 13, Taurine; 14, Glycerol-3-phospahte; 15, Citrate; 16, Glucose; 17, Palmitic acid; 18, Myo-Inositol; 19, Stearic acid; 20, Monopalmitin; 21, Cholesterol.


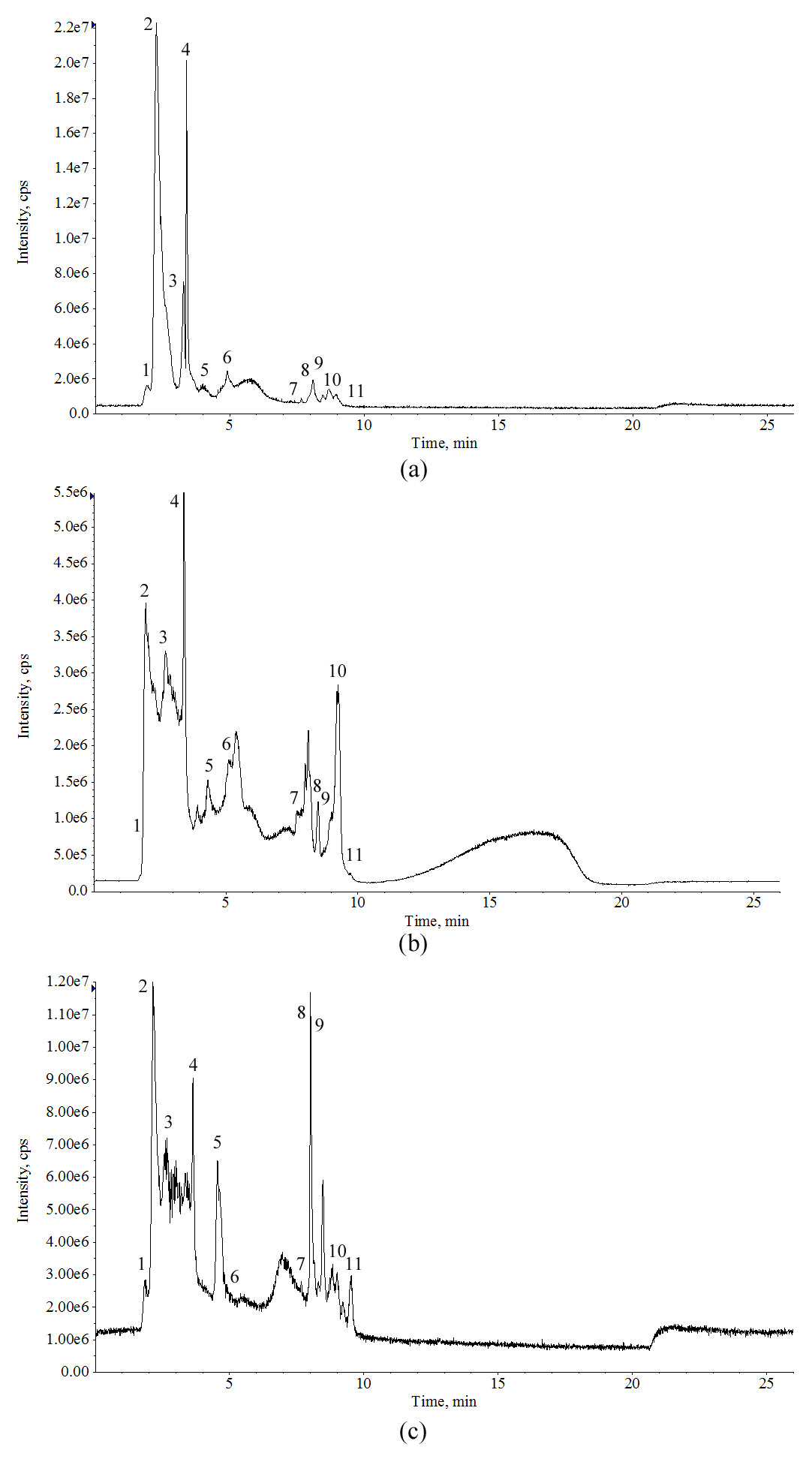


Figure S2: Representative HPLC-QTOF/MS spectra of mice plasma (a), urine (b), and renal cortex (c). Typical compounds are identified and numbered as follows: 1, Cytidine monophosphate; 2, Ribose 5-Phosphate; 3, Nicotinamide; 4, Glucosamine; 5, Glyceric acid; 6, Orotidine; 7, Allantoin; 8, Homocysteine; 9, Inosine; 10, IS; 11, Thymine.
